# *MSLN* Gene Silencing Has an Anti-Malignant Effect on Cell Lines Overexpressing Mesothelin Deriving from Malignant Pleural Mesothelioma

**DOI:** 10.1371/journal.pone.0085935

**Published:** 2014-01-21

**Authors:** Ombretta Melaiu, Justin Stebbing, Ylenia Lombardo, Elisa Bracci, Norihisa Uehara, Alessandra Bonotti, Alfonso Cristaudo, Rudy Foddis, Luciano Mutti, Roberto Barale, Federica Gemignani, Georgios Giamas, Stefano Landi

**Affiliations:** 1 Department of Biology, University of Pisa, Pisa, Italy; 2 Department of Surgery and Cancer, Imperial College London, London, United Kingdom; 3 Second Department of Pathology, Kansai Medical University, Moriguchi-Shi, Osaka, Japan; 4 Department of Endocrinology and Metabolism, Orthopedics and Traumatology, Occupational Medicine, University of Pisa, Pisa, Italy; 5 Laboratory of Clinical Oncology, Vercelli National Health Trust, Vercelli, Italy; National Cancer Institute, NIH, United States of America

## Abstract

Genes involved in the carcinogenetic mechanisms underlying malignant pleural mesothelioma (MPM) are still poorly characterized. So far, mesothelin (*MSLN*) has aroused the most interest. It encodes for a membrane glycoprotein, frequently over-expressed in various malignancies such as MPM, and ovarian and pancreatic cancers. It has been proposed as a diagnostic and immunotherapeutic target with promising results. However, an alternative therapeutic approach seems to rise, whereby synthetic molecules, such as antisense oligonucleotides, could be used to inhibit MSLN activity. To date, such a gene-level inhibition has been attempted in two studies only, both on pancreatic and ovarian carcinoma cell lines, with the use of silencing RNA approaches. With regard to MPM, only one cell line (H2373) has been employed to study the effects of MSLN depletion. Indeed, the knowledge on the role of MSLN in MPM needs expanding. Accordingly, we investigated the expression of *MSLN* in a panel of three MPM cell lines, i.e. NCI-H28, Mero-14, and IstMes2; one non-MPM cell line was used as reference (Met5A). MSLN knock-down experiments on MSLN-overexpressing cells were also performed through silencing RNA (siRNA) to verify whether previous findings could be generalized to a different set of cell cultures. In agreement with previous studies, transient *MSLN*-silencing caused decreased proliferation rate and reduced invasive capacity and sphere formation in *MSLN*-overexpressing Mero-14 cells. Moreover, *MSLN*-siRNA combined with cisplatin, triggered a marked increase in apoptosis and a decrease in proliferation as compared to cells treated with each agent alone, thereby suggesting a sensitizing effect of siRNA towards cisplatin. In summary, our findings confirm that *MSLN* should be considered a key molecular target for novel gene-based targeted therapies of cancer.

## Introduction

Malignant pleural mesothelioma (MPM) is a cancer of the pleural cavity triggered by asbestos exposure. Patients with MPM have a poor prognosis, with overall survival typically ranging between 6 and 13 months. The carcinogenetic mechanisms underlying MPM and the genes involved are still poorly characterized although, so far, *MSLN* has aroused the most interest. The human *MSLN* gene encodes a ∼71 kDa precursor protein of 622 amino acids. The precursor is processed by a removal of 33 N-terminal residues. Moreover, the C-terminal residues departing from Ser598 are replaced with glycosyl-phosphatidyl-inositol (GPI) facilitating the anchoring of the peptide to the cell membrane. Then, the precursor is cleaved at Arg295 by the endoprotease furin into a ∼31 kDa soluble peptide called “megakaryocyte potentiating factor” (from aminoacid Ser34 to Arg286) [1] and a ∼40 kDa GPI-anchored membrane-bound glycoprotein (mature mesothelin, MSLN, starting from Glu296) [2], [3], [4]. It was found that MSLN is present at low levels in a restricted set of normal adult tissues, including the mesothelium, but it is overexpressed aberrantly by several cancers, such as MPM, and pancreatic (PC) and ovarian carcinomas (OC) [5], [6]. Moreover, a soluble form of MSLN (soluble mesothelin related peptide, SMRP) is known, lacking the C-terminal GPI-membrane anchor binding segment [7]. Interestingly, the levels of SMRP are elevated in the sera of MPM, PC, or OC patients but not in patients with other types of cancer or inflammatory diseases, or in healthy controls [8], [9], [10]. Unfortunately, since *MSLN* knock-out mice did not exhibit any adverse pathology, the exact function of MSLN remains unclear [11]. Recent studies highlighted the possible mechanisms by which MSLN could play an active role in cancer progression; it was shown to interact with MUC16 [12], and to activate the p38 pathway, leading to the selective induction of matrix metalloproteinase (MMP)-7 [13]. MSLN could also increase cancer cell survival and proliferation via the activation of the NF-κB signaling pathway [14]. Finally, it was suggested that MSLN could exert its role in the malignant transformation of human cells, through the β-catenin pathway, an important molecule for the epithelial-mesenchymal transition [15].

For all these reasons, MSLN was considered a good target for immunotherapeutic strategies. In fact, it was used to deliver immunotoxins to specific cancer cells [16], [17], [18], [19], [20], [21], or, such as for the case of the monoclonal antibody MORAb-009, to arrest cancer progression by direct inhibition (e.g. disrupting the interaction with MUC16) [22]. Although the use of monoclonal antibodies could provide several advantages (indeed MORAb-009 is currently under clinical trial), target-specific drugs or novel inhibitors (such as antisense oligonucleotides) acting at gene-level could be an alternative for complete inhibition.

To date, direct inhibition of mesothelin with non-immune strategies has been attempted in a very limited number of studies, using silencing RNA (siRNA) approaches. One study, on the Eker (Tsc2 mutant) rat model of hereditary renal cancer, showed tumor growth inhibition following the use of siRNA microspheres designed against Erc, which is considered the rat homologue of *MSLN* [23]. On human cells, one study was carried out on PC cell lines AsPC-1, Capan-1, and Capan-2 [24], whereas another one was performed on cell lines from PC (Miapaca2 and Panc-1) and OC (Skov3 and Ovcar-5) [25]. Overall, MSLN depletion significantly hampered proliferation and colony-forming capability. A decreased viability and invasiveness of PC and OC cell lines were also observed [25]. Moreover, the expression of bcl-2 decreased whilst that of PUMA and Bax increased; at the same time, the activity of caspase-3 increased. Consistently with these observations, an increased apoptotic rate was observed in PC cells and the data were conversely corroborated when MSLN was ectopically over-expressed in HPAC cells, a PC cell line poorly expressing MSLN [24]. With regard to MPM, so far only one cell line (H2373) has been employed to study the effects of MSLN depletion [25]. Indeed, the knowledge on the role of MSLN in MPM should be expanded. Accordingly we investigated the expression of MSLN in a panel of three MPM cell lines, i.e. NCI-H28, Mero-14, and IstMes2; one non-MPM cell line was used as reference (Met5A). We then performed MSLN knock-down experiments in highly expressing MSLN cells, through gene silencing (using silencing RNA, siRNA) to verify whether previous findings could be generalized to a different set of cell cultures, further corroborating the importance of MSLN in the biology of MPM.

## Materials and Methods

### Cell cultures

Three mesothelioma cell lines (Mero-14, IstMes2, and NCI-H28) and one mesothelial non-MPM immortalized cell line (Met5A) were used. Mero-14 [26], and IstMes2 [27] mesothelioma cells had been kindly donated by the Istituto Tumori of Genova (National Research Council, Genoa, Italy). The Met5A mesothelial cells and the NCI-H28 mesothelioma cells had been purchased from the ATCC (American Type Culture Collection) and kindly donated by collaborators of the Pharmaceutical Department of the University of Pisa. Met5A, Mero-14, and NCI-H28 cell lines were verified for their identity, by analyzing the genetic markers reported in the certification. IstMes2 is a locally established cell line. Mero-14, and IstMes2 were cultured in DMEM medium (Lonza, Basel, Switzerland). The NCI-H28 cell line was grown in RPMI 1640 medium (Gibco, Life Technologies, Monza, Italy). The Met5A cell line was grown in Medium199 with HEPES (Life Technologies, Monza, Italy) supplemented with 3.3 nM epidermal growth factor (EGF, Life Technologies, Monza, Italy), 400 nM hydrocortisone (Sigma Aldrich Corp. St Louis, MO, USA), and 870 nM insulin (Life Technologies, Monza, Italy). All the cell lines were cultured with supplement of 10% fetal bovine serum (Sigma Aldrich Corp. St Louis, MO, USA), and 1% Pen-Strep (Lonza, Basel, Switzerland), and maintained at 37°C in a 5% CO_2_–humidified atmosphere (Forma* 311 Direct Heat CO2 Incubator, Thermo Scientific, Waltham, MA, USA).

### RNA isolation and cDNA synthesis

Total RNA was isolated from each cell line with Rneasy Mini kit (Qiagen MI, Italy), according to the standard protocol. In order to remove possible contaminating genomic DNA, the extracted RNA was treated with DNAse buffer (Sigma Aldrich Corp. St Louis, MO, USA). Concentration and purity of cleaned-up RNA were determined with a spectrophotometer (SmartSpec 3000, Bio-Rad Laboratories, Hercules, CA). The integrity of total RNA was verified by electrophoresis on ethidium bromide agarose gel, inspecting the 18S and 28S ribosomal RNA bands. Reverse transcription (RT) was performed with the *iSCRIPT cDNA Synthesis Kit* using 1µg of total RNA in a final volume of 20µl (Bio-Rad Laboratories, Hercules, CA).

### Quantitative Real-Time PCR (RT-qPCR)

Pre-designed TaqMan probes (Life Technologies, Monza, Italy) were employed. For the TaqMan assay, the reaction mixture consisted of 2 µl of cDNA template, 7 µl of deionized H_2_O, 1 µl of specific TaqMan Assay probe and primer mixture, and 10 µl of TaqMan® Gene Expression Master Mix (Life Technologies, Monza, Italy). The thermal cycling conditions were: 15 min at 95°C followed by 15 s at 95°C and 60 s at 60°C (40 cycles). TaqMan ID assays are reported in Table S1. Seven housekeeping genes, *GAPDH*, *HPRT1*, *B2M*, *RPLP0*, *TBP, GUSB* and *PPIA*, were tested for stability, to be used as reference. The three most stable genes (*RPLP0*, *HPRT*, and *TBP*) were determined based on the average M and the pair-wise variation values, calculated with the tool geNorm [28].

### Chemicals

The drugs were dissolved in DMSO at the final concentration of 10 mM. Imatinib was purchased from Cayman Chemical (Michigan, USA) and used in the range of 5–25 µM; Gemcitabine was obtained from Sigma Aldrich Corp. (St Louis, MO, USA) and used in the range of 1–10 µM; Cisplatin, kindly donated by Prof. Justin Stebbing (Imperial College, London), was used in the range 1–25 µM. The following antibodies were used: MSLN mouse monoclonal (Santa Cruz); β-actin mouse monoclonal (Abcam), p53 mouse monoclonal (Santa Cruz); pERK, mouse polyclonal (Abcam); PARP rabbit polyclonal (Cell Signaling); pAKT rabbit polyclonal (Abcam); ERK1-2 rabbit polyclonal (Abcam); Secondary HRP (horseradish peroxidase)-conjugated goat anti-rabbit IgG and goat antimouse IgG antibodies were from GE Healthcare. The expression plasmid pcDNA3.1 encoding for *MSLN* (aa 360-2230) was kindly donated by Dr. Uehara (Kansai Medical University, Japan); the empty vector pcDNA3.1, employed as control, was donated by Dr. Giamas (Imperial College, London).

### siRNA and plasmid transfections

siMSLN-1 and-2 were purchased from Qiagen (Qiagen, S.p.A, Milano, Italy). The “AllStars Negative Control siRNA” (SI03650318) was used as non-targeting control (siRNA-Ctrl). siRNA oligonucleotides were re-suspended in the provided buffer at a final stock concentration of 20 µM. siRNA transfection was performed with the HiPerfect transfection reagent (Qiagen, S.p.A, Milano, Italy), according to the manufacturer’s instructions. Plasmid transfections were performed using the FuGENE® Transfection Reagent (Promega Corp. Madison, Wisc., USA) in penicillin/streptomicine-free DMEM for 24 hs according to manufacturer's instructions.

### Protein extraction and Western Blotting

Cell pellets were suspended in ice-cold RIPA buffer containing protease and phosphatase inhibitors (Sigma Aldrich Corp. St Louis, MO, USA). The extracts were then clarified by centrifugation at 15000 rpm for 15 min at 4°C, and the protein concentration was determined with the Bradford reagent (Bio-Rad Laboratories, Hercules, CA) and spectrophotometric analysis. Lysates were incubated in 5x sodium dodecyl sulfate (SDS) sample buffer (5 min, 95°C). An amount of 10-30 µg of proteins for each sample was loaded onto 8–15% SDS polyacrylamide gel. Proteins were then transferred onto a nitrocellulose membrane. The membrane was blocked for 1h with non-fat dry milk in TBS containing 0.05% Tween 20, washed, and successively incubated with different primary antibodies for 12h at 4°C. The membranes were then washed three times for 10 min and incubated with the HRP-conjugated secondary antibody for 1h at room temperature (RT). After a thorough washing, the blot was exposed to ECL (GE Healthcare, NJ) followed by autoradiography. The intensity of the bands was quantified using Image J software (NIH, Bethesda, MD).

### Sulphorhodamine (SRB) assay

Cells were seeded in 96 well plates at a density of 3×10^3^. The next day, (day 0), one plate was assessed. The remaining plates were tested at 2-day intervals for a total of 6–8 days. Cells were fixed with 100 µL per well of ice-cold 40% (vol/vol) TCA (Sigma Aldrich Corp. St Louis, MO, USA) gently added on top of the medium overlaying the cells. The plates were then incubated for 60 min at 4°C. Wells were rinsed five times with tap water and then stained with 0.4% SRB solution (100 µl stain/well; Sigma Aldrich Corp. St Louis, MO, USA) for 30 min at RT. After staining, SRB solution was removed, unbound dye was removed by washing five times with 1% acetic acid solution and left to air dry. The bound SRB dye was then solubilized by adding unbuffered Tris-base solution (100 µl/well), and plates were placed on a plate shaker for 10 min at room temperature. Plates were then read at OD 492 nm, using a microplate reader.

### 3D Overlay Culture on Matrigel

Thawed Matrigel (BD Bioscience) in a volume of 70 µl/well was added into each of the wells of the eight-well glass slide chambers (Thermo Scientific), and spread to form a 1-mm thick bed. Matrigel was left to solidify at 37°C for 15 min. Then, cells (1×10^3^/well) were plated in medium containing 2% Matrigel and allowed to grow in a 5% CO_2_ humidified incubator at 37°C.

### Flow cytometry (FACS)

After treatments, 1×10^5^ cells were collected, washed in phosphate-buffered saline (PBS), pelleted by centrifugation and fixed in 70% ethanol. Immediately prior to staining, the cells were washed twice in PBS and suspended in PBS containing 50 µg/ml of RNAse A (Qiagen, S.p.A, Milano, Italy). The cells were stained with propidium iodide (final concentration 100 µg/ml) for at least 1 h at 4°C and analyzed using a LSR II flow cytometer (BD Biosciences). The percentage of cells in subG1, G0/G1, S and G2/M phases were determined from >10,000 cells using the FACSDiva 6.0 software (BD Biosciences).

### Caspase - Glo® 3/7 assay

Caspase-3/7 activation was measured using the Caspase-Glo 3/7 Luminescence Assay (Promega Corp. Madison, Wisc., USA) according to the manufacturer’s instructions. In a 6 well plate 3×10^5^ cells were incubated. The day after, the cells were treated with siRNAs, both with and without drugs, for 48 h. Then, the cells were collected by trypsinization, and approximately 15×10^3^ cells were transferred in a 96-well white plate. Caspase-3/7-Glo reagent was added, and the samples were incubated at 37°C for 1 h. The luminescence that is proportional to the caspase 3/7 activities was determined by luminometer (Tecan Sunrise, Austria GMBH).

### Transwell Cell Invasion Assay

About 5×10^4^ cells in 200 µl of α-MEM (Gibco, Life Technologies, Monza, Italy) were plated in the Matrigel-coated upper chambers of the 24-well Transwell invasion assay plate (Corning, NY 14831 USA). Plates were incubated at 37°C for 48 h. Cells in the lower chamber (including those attached to the lower surface of the membrane) were fixed in 4% paraformaldehyde (VWR, Milan Italy), stained with DAPI (Lonza, Basel, Switzerland), and counted with fluorescence microscope (Metamorph – Axiovert microscope).

### Wound-Healing Assay

About 25×10^3^ cells were seeded in a 12-well plate and, after 24 h, transfected with siMSLN-1. A linear scratch in the confluent cell monolayer was made with a sterile pipette tip after 12 h (time optimized following preliminary trials) following siRNA transfections. Then, cells were rinsed and incubated in full medium. Finally, cells were inspected and fixed with paraformaldehyde after 36 h, following the scratch. Cells were stained with crystal violet 0.1%solution (dissolved in 20% ethanol) to enhance contrast and photographed with a phase-contrast microscope at 10X magnification. The migration was then evaluated on the images, and measured with Image J software.

### Statistical analyses

The measurements of gene expression performed on cell lines, and the results obtained from the *in vitro* assays were statistically evaluated using a two-tailed Student’s t-test. The effects of the combination of treatments (drugs +/– siRNAs) were evaluated with a multifactor analysis of variance (MANOVA) model. The statistics were performed with the software R (http://www.r-project.org/) and Statgraphics Centurion XV (StatPoint, Inc.).

## Results

### *MSLN* expression in MPM cell lines

**Figure 1:**
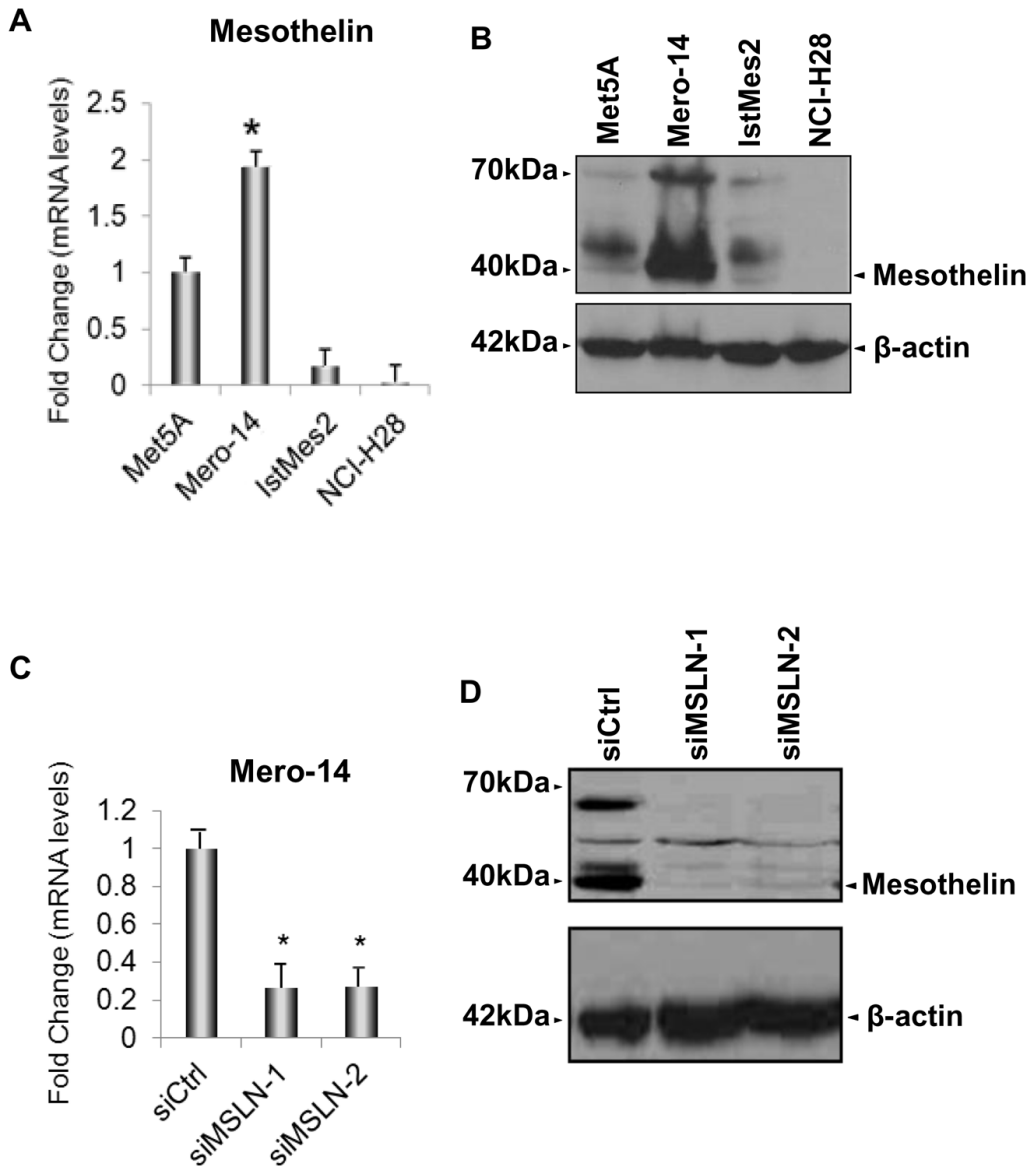
Expression levels of *MSLN* in human MPM cell lines and Met5A. ***A.*** RT-qPCR showing the mRNA expression levels of *MSLN* measured on MPM cell lines and related to Met5A cells (set to 1). *RPLP0*, *HPRT*, and *TBP* were used for normalization. Error bars show the standard error of the mean (SEM) from three independent experiments, each performed in triplicate. Mero-14 cells showed the highest expression levels of *MSLN* (P = 0.02). ***B.*** The protein levels of MSLN in Met5A, Mero-14, IstMes2, and NCI-H28 cells. β-actin was used as reference. The protein levels were confirmed by two independent experiments. MSLN is shown as a band at 40 kDa. ***C.*** RT-qPCR showing the endogenous mRNA expression levels of *MSLN* in Mero-14 cells, related to their own siCtrl (set to 1). *RPLP0*, *HPRT*, and *TBP* were used for normalization. Error bars are SEM, from three independent experiments, each performed in triplicate. The siRNA chosen for the analysis is: siMSLN-1 (40 nM; *P = 0.002) active on Mero-14 cells. ***D.*** Protein levels of MSLN (shown as a band at 40 kDa) after depletion with siMSLN-1 and -2 (40 nM). β-actin was used as reference. The protein levels were confirmed by three independent experiments.

The expression level of *MSLN* was screened in Mero-14, IstMes2, and NCI-H28 human MPM cell lines. Met5A, a non-malignant immortalized cell line, was also screened and used as reference. The expression of *MSLN* mRNA in Mero-14 cells was higher than in Met5A (Figure 1A). Western blotting supported these data, showing high MSLN levels in Mero-14 cells (Figure 1B) and low levels in Met5A. Similar to mRNA expression, IstMes2 had lower levels of MSLN than Met5A.No levels of MSLN were observed for NCI-H28. Thus, two different silencing-RNAs (siRNAs) were assayed: siMSLN-1 and siMSLN-2 for *MSLN* in Mero-14 cells, (Table S2; Figure 1C). For further experiments, siMSLN-1 was employed given its better performance in gene silencing (>95% for siMSLN-1 Figure 1D).

### Role of *MSLN* in cellular growth

**Figure 2:**
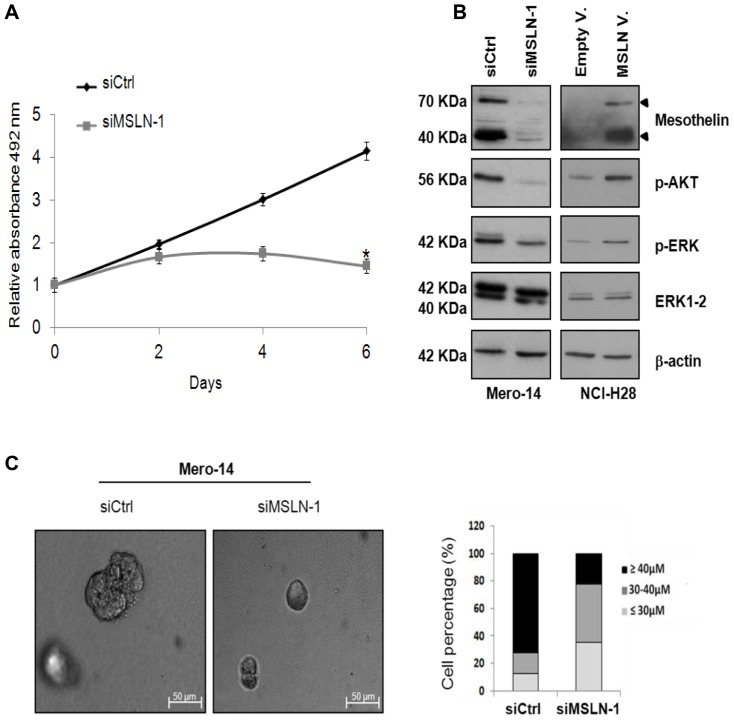
Role of MSLN in cellular growth. ***A.*** SRB proliferation assay in Mero-14 cells treated with 40 nM of the siCtrl or siMSLN-1 (*P  =  <10^−4^). Error bars represent SEM of three independent experiments, each performed in quadruplicate. ***B.*** Western blotting analysis of MSLN, p-AKT, p-ERK, and ERK1-2, on Mero-14 cells treated with siCtrl, or siMSLN-1, and on NCI-H28 cells transfected with an empty vector (pcDNA3.1) or a plasmid overexpressing *MSLN* (pcDNA3.1-MSLN). β-actin was used as reference. The protein levels were confirmed with three independent experiments. ***C*** **.** The picture represents the phase contrast microscopy of Mero-14 cells cultured in 3D Matrigel-overlay chambers after silencing of *MSLN* (siMSLN-1 at 40 nM). Magnification 10X. Two different experiments were performed, each in triplicate. The percentages of Mero-14 cells classified according to the dimensions of the spheres following treatments with siCtrl or siMSLN-1 in 3D Matrigel-overlay chambers were also reported. **Legend to** **figure 2**: Gray line =  cells treated with siMSLN-1; Dark line =  cells treated with siCtrl.

The effect of *MSLN* silencing on cellular growth was evaluated by performing two different assays: the SRB assay and the 3D Matrigel-overlay model. The first facilitates the assessment of the number of cells grown in a bi-dimensional context at a given time [29], whilst the latter, facilitates the evaluation of the dimension and shape of spheroids formed in a three-dimensional context. Following the administration of siMSLN-1, a significant reduction (p < 0.05) in the proliferation rate was observed for Mero-14 cells, starting from the third day of treatment, as compared to cells treated with control siRNA (siCtrl), reaching a 86% decrease at day 6 (Figure 2A). This result was also corroborated by a reduced expression of the phosphorylated forms of AKT and ERK, pAKT and pERK being markers of proliferation [30] (Figure 2B). When *MSLN* was transiently over-expressed in the non-*MSLN* expressing NCI-H28 cells, increased levels of pAKT and pERK were observed, confirming a link between *MSLN* expression and proliferation (Figure 2B). The silencing of *MSLN* in Mero-14 cells was also associated with smaller and uniform spheres (mean  =  34.4 µm±3.11), as compared to the cells treated with siCtrl (mean  =  52.5 µm, ±7.65, p<10^−6^). About 72.5% of siCtrl-treated spheres and only 22.5% of siMSLN-1-treated spheres measured > 40 µm (Figure 2C). Thus, Mero-14 cells following MSLN silencing showed a low proliferation rate and a reduced capacity of forming spheroids.

### Role of *MSLN* in cell cycle progression and apoptosis

**Figure 3:**
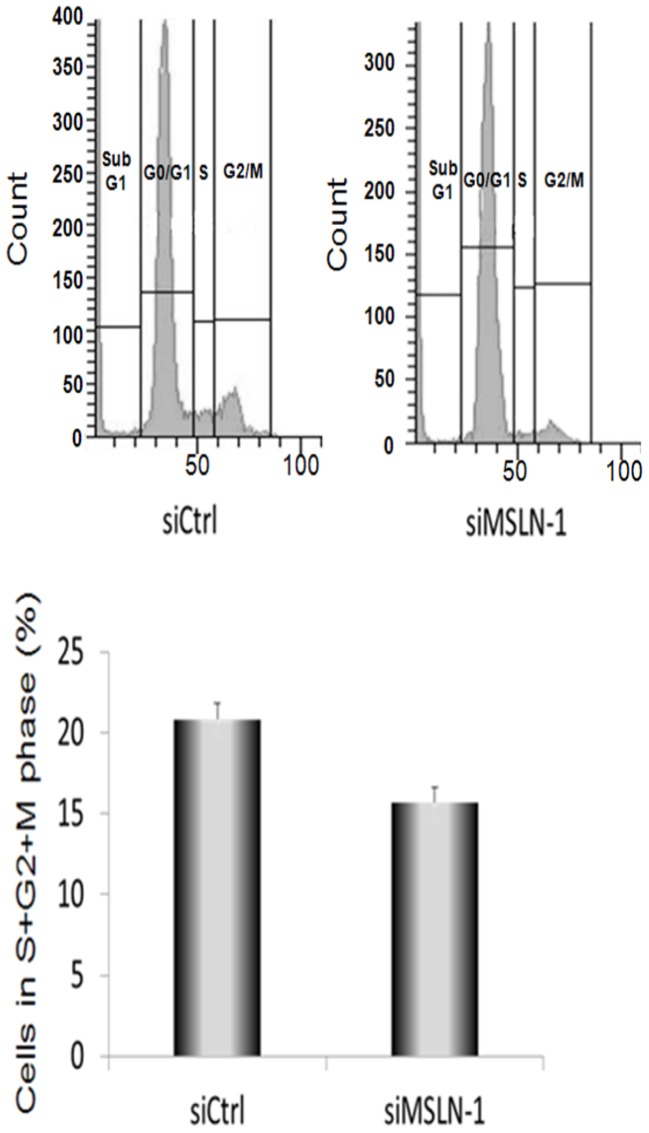
Progression of cells through the cell cycle following flow cytometry analysis. The graph shows the percentage of Mero-14 cells in phase S+G2+M treated with 40 nM of the siCtrl or siMSLN-1. The S+G2+M phase of the cell cycle was slightly reduced following the treatment with siMSLN-1 (*P =  0.033). Error bars represent SEM of six independent experiments.

To examine the effects of *MSLN* silencing on cell cycle progression, Mero-14 cells were treated with siCtrl and siMSLN-1, for 72 h and analyzed with flow cytometry. A statistically significant decreased share of Mero-14 cells in S+G2+M-phases was observed following *MSLN* silencing as compared to controls (Figure 3). The reduction (standardized for siCtrl) was approximately by 25%. Measured at 48 h after siRNA transfection, the Mero-14 cell line did not show any changes in the activities of apoptosis markers: caspase-3 and -7.

### Role of *MSLN* in migration and invasion

**Figure 4:**
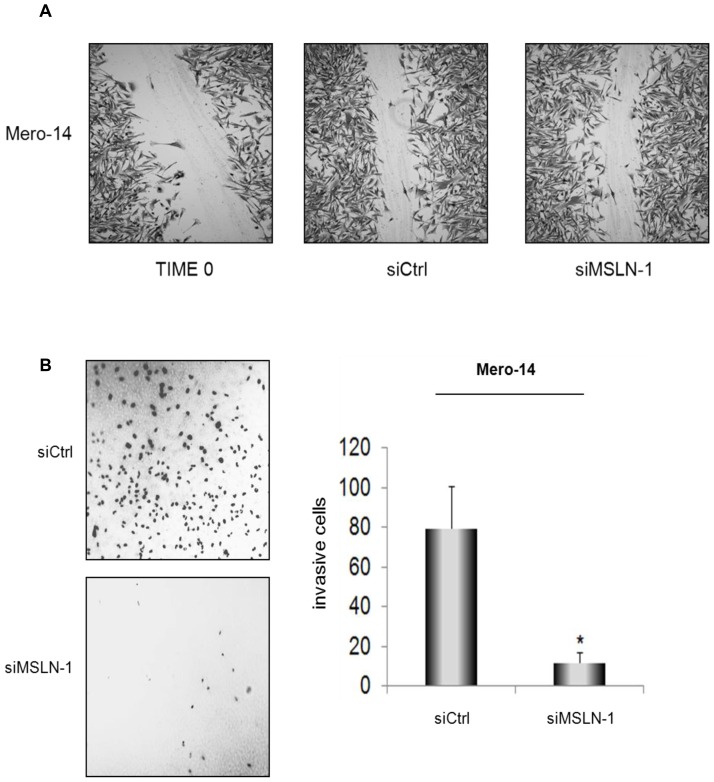
Role of MSLN in cellular migration and invasion. ***A*.** No effects observed in the wound-healing assay, following siRNA transfections. Confluent monolayers of Mero-14 cells transfected with 40 nM of siCtrl, or siMSLN-1, respectively. Two different experiments were carried out, each performed in triplicate. ***B*** **.** Trans-well cell invasion assay on Mero-14 cells transfected with 40 nM of the siCtrl (top), or siMSLN-1 (bottom). Pictures were taken using a fluorescence microscope at 10X magnification and are reported as negative of the originals to enhance the contrast between the background and the DAPI-stained cells. The bar chart shows the average of invasive cells (error bars represent SEM of two independent experiments, each done in triplicate, *P  =  0.0044).

The effect of gene silencing on cellular migration was evaluated using the wound-healing assay (assessing the repopulation of a scratched area in a plate) [31].The invasiveness was measured using the trans-well assay (assessing the number of cells passing through the pores of the membrane) [32]. No statistically significant differences in migration parameters were observed in Mero-14 following the siRNA treatments (Figure 4A). However, Mero-14 cells showed a statistically significant reduced invasion, as compared to controls, at 48 h after *MSLN* silencing (Figure 4B).

### Role of *MSLN* in cellular growth following treatments with chemotherapeutic drugs

**Figure 5:**
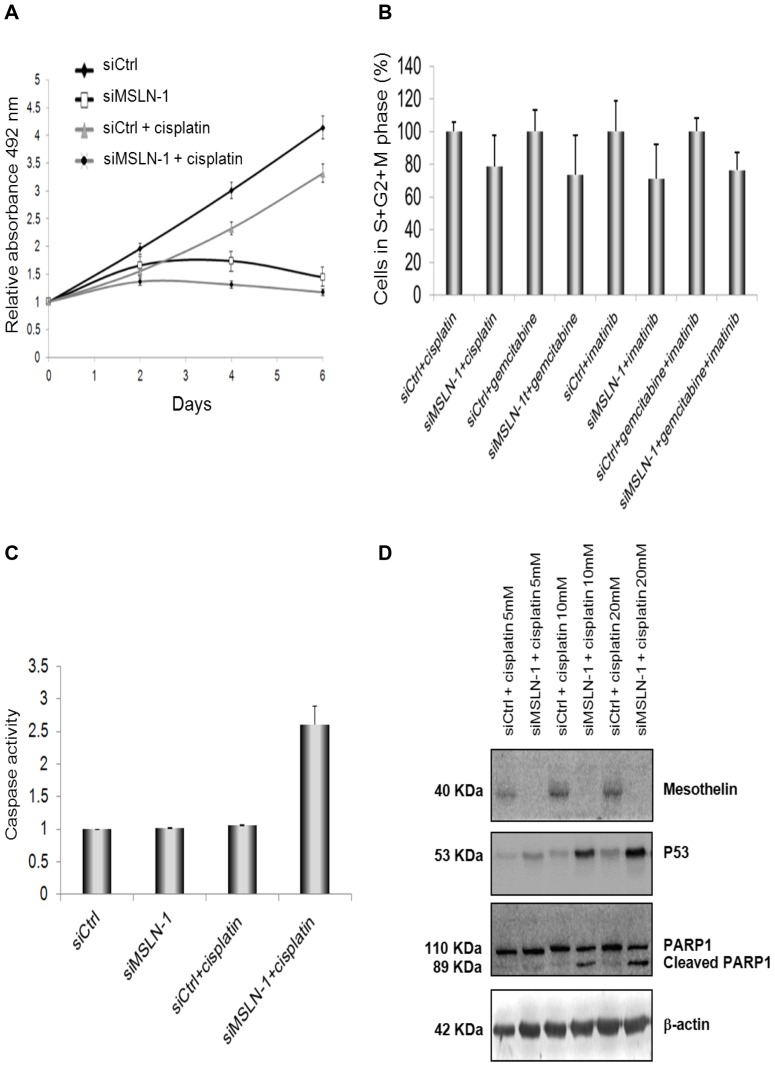
Role of MSLN in cellular growth, cell cycle progression and apoptosis, following treatment with chemotherapeutic drugs. *A*. Proliferation assay in Mero-14 cells. The graph shows the effect of the treatments with 5 µM cisplatin and 40 nM siMSLN-1, used as single agents or in combination. On day 6, MANOVA shows a statistically significant effect both for cisplatin (P = 0.0168) and siMSLN-1 (P<10^−4^) in reducing proliferation. However, the interaction term for the effect of both agents in combination is not statistically significant (P = 0.145). Error bars represent SEM of three independent experiments, each performed in quadruplicate. *B*. Flow cytometry analysis. The graph shows the percentage of cells in phase S+G2+M in Mero-14 cells treated with 40 nM of the siCtrl or siMSLN-1 in combination with imatinib (25 µM) or gemcitabine (1 µM) (alone) or imatinib+gemcitabine (10 µM and 1 µM, respectively). The transfection with siMSLN-1 was accompanied with a marked decrease of cells in S+G2+M phase, as compared with the respective cultures transfected with siCtrl, irrespectively of the drugs employed (P =  0.00033). Error bars represent SEM of two independent experiments. *C*. Caspase activity measured on Mero-14 cells transfected with 40 nM of siCtrl, or siMSLN-1, with or without cisplatin 5 µM. A marked increase in apoptosis is observed when siMSLN-1 and cisplatin are administered together, compared to cultures treated with cisplatin and transfected with siCtrl (*P = 0.018), suggesting a synergistic effect. Error bars represent SEM of three independent experiments, each performed in triplicate. *D*. Western blotting analysis of MSLN, p53, and PARP under different combinations of siRNAs and cisplatin (at 5, 10 and 20 µM). β-actin was used as reference. The protein levels were confirmed with three independent experiments. Legend to figure 5: Dark line: cells trated with siCtrl; gray line and triangles: cells treated with siCtrl plus cisplatin; gray line and dark spots: cells treated with siMSLN plus cisplatin; dark line and white spots: cells treated with siMSLN-1.

After 6 days of treatment, cisplatin used as a single agent caused a 26% reduction (not statistically significant) in the proliferation rate of Mero-14 cells. Then, the effect of siMSLN-1 was evaluated in combination with cisplatin. When the two agents were used in combination, the growth was completely inhibited (p<0.05, Figure 5A). Moreover, the addition of siMSLN-1 in cultures treated with imatinib or gemcitabine (each as a single agent) or imatinib+gemcitabine caused further reductions in proliferation. However, the effect of siMSLN-1 was not statistically significant, in contrast to cultures treated with the two chemotherapeutic drugs together with siCtrl (p = 0.21, p = 0.38, and p = 0.17, respectively).

### Role of *MSLN* in cell cycle progression and apoptosis following treatments with chemotherapeutic drugs

Following flow cytometry analysis, Mero-14 cells treated with siMSLN-1 in combination with cisplatin or imatinib or gemcitabine (each as a single agent) or imatinib+gemcitabine, showed a statistically significant decreased share of cells in S+G2+M phase, as compared to their respective cultures where siMSLN-1 were replaced with siCtrl (Figure 5B). This finding further confirmed the activity of siMSLN-1 in slowing the progression through cell cycle.

In treatments where siRNA was combined with chemotherapeutic drugs, activities of caspases-3 and -7 were measured as markers for apoptosis. The addition of siMSLN-1 in cultures treated with imatinib or gemcitabine (each as a single agent) or imatinib+gemcitabine was not associated with an increased rate of apoptosis as compared to cultures treated with the chemotherapeutic drugs together with siCtrl. Interestingly, a synergistic effect was observed when cisplatin was used in combination with siMSLN-1. In fact, siMSLN-1 or cisplatin alone did not induce apoptosis, whereas they markedly (and in a statistically significant way) induced increased apoptosis rates when used together (Figure 5C). This observation was further corroborated by the induction of p53 and by the cleavage of PARP, both additional markers for apoptosis (Figure 5D). The effect was dose-dependent and visible from 5 µM of cisplatin.

## Discussion

The present work provides evidence on the importance of MSLN for cell growth and invasiveness in MPM. The transient MSLN-silencing caused a decrease in the proliferation rate of the MSLN-overexpressing cell line Mero-14. These data are in agreement with those observed on PC cells [24]. Similar findings were also reported by Wang et al. in the MSLN-overexpressing MPM cell lines H2373 [25]. As with the H2373 MPM cells, the substantial arrest of the proliferation rate observed in the Mero-14 cells was underlined by the shift of the phosphorylation status of AKT and ERK (used as a marker of proliferation). The results on MPM cells were in agreement with the findings observed in PC and OC cells [25], suggesting that all the MSLN-expressing cancer cells show a significant loss of viability upon MSLN depletion. In addition to the reduced proliferation, Mero-14 cells also showed a reduced capacity of sphere formation in a three-dimensional context. Concerning the cell cycle, a significant increase (50%) of MPM H2373 cells in the S-phase was observed portraying a blockade in progression from S to G2 phase [25]. The results obtained in Mero-14 cells were different, since a reduction of cells in S-phase was observed, paralleling an increase of cells in G1 phase. The differences could be ascribed to the different methods of siRNA administration (electroporation in H2373 versus chemical transfection in Mero-14) involving different time of observation (48 versus 72 hours, respectively). However, the overall decrease of cells in G2/M was consistent in both cell lines. Moreover, a significant reduction in invasiveness was observed in both Mero-14 and H2373 cells in the trans-well assay. With regard to apoptosis, no assays were reported for H2373. In general, MPM cell lines are quite refractory to undergo apoptosis and this was also observed in Mero-14 cells after MSLN depletion or a treatment with cisplatin. By contrast, MSLN silencing was able to promote apoptosis in PC AsPC-1, Capan-1, and Capan-2 cells [24]. However, *MSLN* depletion triggered a marked increase in apoptosis in Mero-14 cells when used in combination with cisplatin, thereby suggesting a synergistic effect. In Mero-14 cells, the activation of caspases-3 and 7 was associated with the induction of p53 and with the cleavage of PARP, both markers of a pro-apoptotic activity.

In summary, paralleling previous studies, our findings confirm that *MSLN* should not be regarded only as an interesting diagnostic marker for MPM or a promising target for immunotherapies. Despite the limited knowledge on the biological role of MSLN in normal and cancer cells, MSLN should also be considered a key molecular target for novel gene-based targeted therapies of cancer.

## Supporting Information

Table S1 **Genes analysed for their mRNA expression in the present work.** The table reports, in the order, the gene name, the gene bank ID code, the ID numbers of the TaqMan® assays, the melting temperatures (in C°), and the lengths of the amplicons.
(DOC)

Table S2 **Silencing-RNAs tested in the present work.** The table reports, in the order, the targeted gene, the siRNAs codes, and the targeted sequences.
(DOC)
