# Correction: *MSLN* gene silencing has an anti-malignant effect on cell lines overexpressing mesothelin deriving from malignant pleural mesothelioma

**DOI:** 10.1371/journal.pone.0180317

**Published:** 2017-06-22

**Authors:** Ombretta Melaiu, Justin Stebbing, Ylenia Lombardo, Elisa Bracci, Norihisa Uehara, Alessandra Bonotti, Alfonso Cristaudo, Rudy Foddis, Luciano Mutti, Roberto Barale, Federica Gemignani, Georgios Giamas, Stefano Landi

In Fig 4A, the incorrect image is used for siMSLN-1. Please see the correct Fig 4 here.

**Fig 4:**
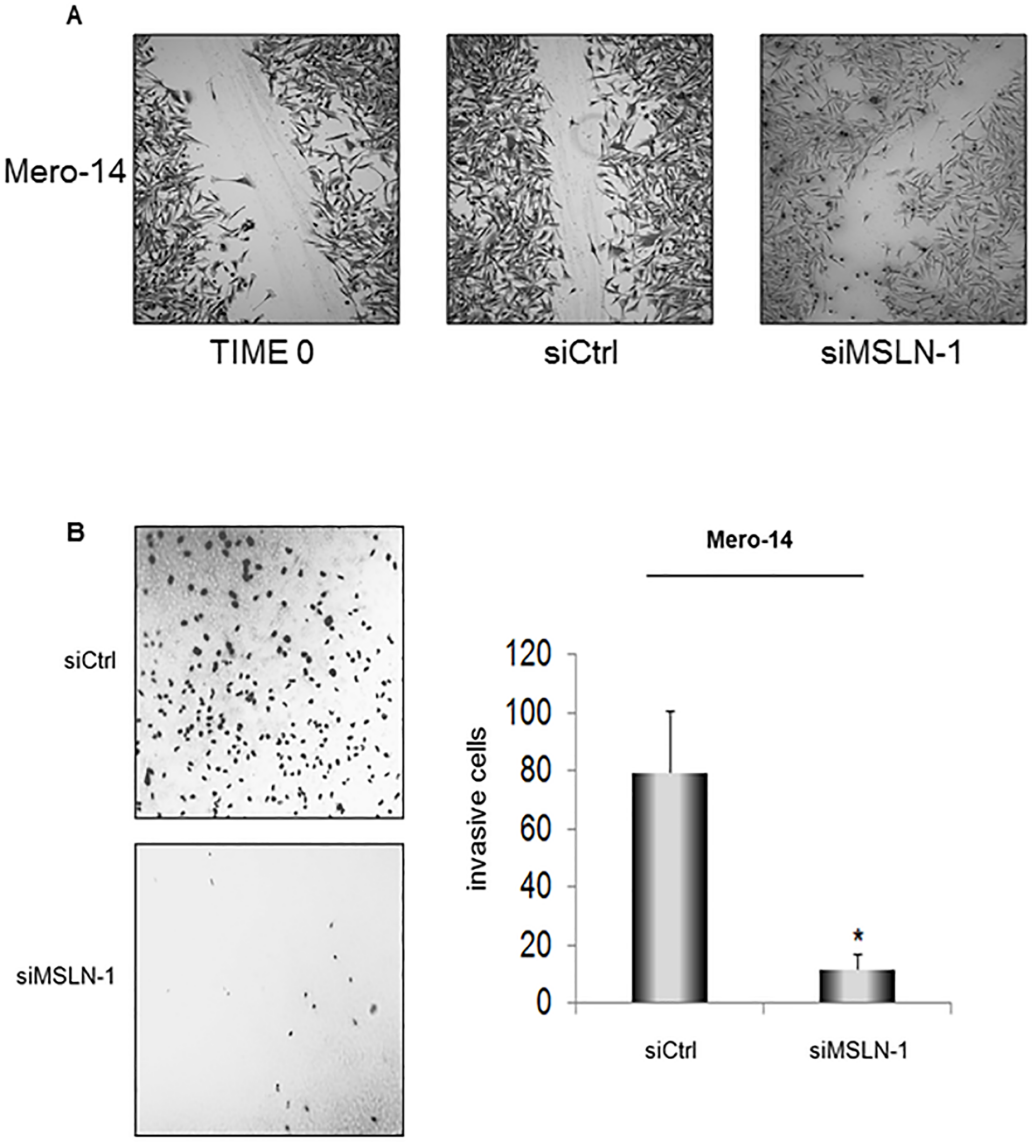
Role of MSLN in cellular migration and invasion. ***A*.** No effects observed in the wound-healing assay, following siRNA transfections. Confluent monolayers of Mero-14 cells transfected with 40 nM of siCtrl, or siMSLN-1, respectively. Two different experiments were carried out, each performed in triplicate. ***B*.** Trans-well cell invasion assay on Mero-14 cells transfected with 40 nM of the siCtrl (top), or siMSLN-1 (bottom). Pictures were taken using a fluorescence microscope at 10X magnification and are reported as negative of the originals to enhance the contrast between the background and the DAPI-stained cells. The bar chart shows the average of invasive cells (error bars represent SEM of two independent experiments, each done in triplicate, *P = 0.0044).
