# Supplementary material for: MSLN Gene Silencing Has an Anti-Malignant Effect on Cell Lines Overexpressing Mesothelin Deriving from Malignant Pleural Mesothelioma
Source: PLoS One. 2014 Jan 21;9(1):e85935. doi: 10.1371/journal.pone.0085935 (PMC3897543; doi:10.1371/journal.pone.0085935)
Supplement: Table S1 — Genes analysed for their mRNA expression in the present work. The table reports, in the order, the gene name, the gene bank ID code, the ID numbers of the TaqMan® assays, the melting temperatures (in C°), and the lengths of the amplicons. (DOC) [file pone.0085935.s001.doc]

**Supplementary material**

**Table S1.** **Genes analysed for their mRNA expression in the present work.**

The table reports, in the order, the gene name, the gene bank ID code, the ID numbers of the TaqMan® assays, the melting temperatures (in C˚), and the lengths of the amplicons.

| **Gene ID** | **NM_code** | **Assay ID** | **T_m_** | **bp** |
| --- | --- | --- | --- | --- |
| *MSLN* | NM_005823 | Hs00245879_m1 | 60 | 65 |
| *PPIA* | NM_021130 | Hs99999904_m1 | 60 | 122 |
| *GAPDH* | NM_002046 | Hs99999905_m1 | 60 | 81 |
| *B2M* | NM_004048 | Hs00984230_m1 | 60 | 96 |
| *GUSB* | NM_000181 | Hs00939627_m1 | 60 | 91 |
| *TBP* | NM_003194 | Hs00427620_m1 | 60 | 91 |
| *HPRT* | NM_000194 | Hs01003267_m1 | 60 | 72 |
| *RPLP0* | NM_053275 | Hs99999902_m1 | 60 | 105 |
