# Supplementary material for: MSLN Gene Silencing Has an Anti-Malignant Effect on Cell Lines Overexpressing Mesothelin Deriving from Malignant Pleural Mesothelioma
Source: PLoS One. 2014 Jan 21;9(1):e85935. doi: 10.1371/journal.pone.0085935 (PMC3897543; doi:10.1371/journal.pone.0085935)
Supplement: Table S2 — Silencing-RNAs tested in the present work. The table reports, in the order, the targeted gene, the siRNAs codes, and the targeted sequences. (DOC) [file pone.0085935.s002.doc]

**Supplementary material**

**Table S2.** **Silencing-RNAs tested in the present work.**

The table reports, in the order, the targeted gene, the siRNAs codes, and the targeted sequences.

| Target Gene | siRNAs | Gene target sequence |
| --- | --- | --- |
| *MSLN* | siMSLN-1 | CTGGACGTCCTAAAGCATAAA |
| *MSLN* | siMSLN-2 | CTCGCGCTCAGTAAACGGGAA |
